# Development of a clinical scoring system to predict response to low-intensity shock wave therapy in clinically diagnosed vasculogenic erectile dysfunction: a prospective, non-randomized, single-arm, interventional study

**DOI:** 10.1093/sexmed/qfag025

**Published:** 2026-05-04

**Authors:** Hakan Anıl, Ali Yıldız, Ayşe Nur Topuz, Mehmet Vehbi Kayra, Sinan Sözütok, Adem Altunkol, Ergün Alma, Mustafa Topuz, Yıldırım Bayazıt

**Affiliations:** Department of Urology, University of Health Sciences, Adana City Training and Research Hospital, Adana, 01230, Turkey; Department of Urology, Faculty of Medicine, Istanbul Okan University, Istanbul, 34959, Turkey; Department of Family Medicine, Cukurova University, Adana, 01130, Turkey; Department of Urology, Baskent University School of Medicine, Adana Dr. Turgut Noyan Medical and Research Center, Adana, 01250, Turkey; Department of Interventional Radiology, Medical Park Seyhan Hospital, Adana, 01140, Turkey; Department of Urology, University of Health Sciences, Adana City Training and Research Hospital, Adana, 01230, Turkey; Department of Urology, University of Health Sciences, Adana City Training and Research Hospital, Adana, 01230, Turkey; Department of Cardiology, Faculty of Medicine, Istanbul Aydın University, Istanbul, 34295, Turkey; Department of Urology, Medical Park Seyhan Hospital, Adana, 01140, Turkey

**Keywords:** erectile dysfunction, low-intensity extracorporeal shock wave therapy, predictive factors, clinical scoring system, flow-mediated dilation

## Abstract

**Background:**

Erectile dysfunction (ED) is a prevalent condition, with vasculogenic ED being the most common subtype, primarily related to endothelial dysfunction and cardiovascular risk factors. Low-intensity extracorporeal shock wave therapy (Li-ESWT) has emerged as a promising non-invasive treatment option. However, predictors of treatment response remain poorly defined.

**Aim:**

To identify clinical and vascular predictors of treatment success following Li-ESWT in patients with vasculogenic ED and to develop a novel, practical, and non-invasive scoring system to predict therapeutic response.

**Methods:**

This prospective study included 219 men aged 18-80 years with vasculogenic ED between January 2024 and January 2025. All patients underwent Li-ESWT (18 000 pulses over 3 weeks). Clinical and vascular parameters, including age, ED duration, body mass index, presence of cardiovascular risk factors, diabetes mellitus (DM), carotid intima-media thickness (cIMT), flow-mediated dilation rate (FMD), and prior phosphodiesterase type 5 inhibitor (PDE5i) response, were recorded. Treatment success at 6 months was defined as an increase of ≥1 point in the erection hardness score or ≥5 points in the International Index of Erectile Function-5 EF.

**Outcomes:**

The primary outcome was treatment success at 6 months after Li-ESWT. Secondary outcomes included the development and validation of a predictive scoring system.

**Results:**

Treatment success rate was 66.2% (145/219). Independent predictors of treatment success were absence of DM (odds ratio [OR] = 2.67, *P* = .012), ED duration <36 months (OR = 2.23, *P* = .026), cIMT <0.8 mm (OR = 2.04, *P* = .042), prior PDE5i benefit (OR = 2.47, *P* = .016), FMD ≥5% (OR = 2.57, *P* = .012), age <65 years (OR = 2.28, *P* = .032), and presence of cardiovascular risk factors (OR = 2.23, *P* = .036). A scoring system incorporating these 7 variables achieved an area under the curve of 0.819 (95% confidence interval [CI] 0.762-0.877). Using a cut-off of 4.5 points, the sensitivity was 73% and the specificity was 77% (*P* < .001).

**Clinical Implications:**

This scoring system may help clinicians identify patients most likely to benefit from Li-ESWT, optimize patient selection, and improve individualized treatment strategies in vasculogenic ED.

**Strengths & Limitations:**

The study is strengthened by its prospective design, relatively large sample size, and inclusion of vascular function parameters (FMD, cIMT). Limitations include the lack of external validation and the absence of penile Doppler ultrasound confirmation in all patients.

**Conclusion:**

This study identified key demographic and vascular predictors of Li-ESWT response and introduces a novel, non-invasive clinical scoring system with strong predictive accuracy. This tool may enhance treatment personalization and support clinical decision-making in the management of ED.

## Introduction

Erectile dysfunction (ED) is defined as the inability to achieve or maintain an adequate erection during sexual intercourse.^1^ Its prevalence is increasing, particularly in men over 40. It presents as a symptom, rather than a disease, to clinicians. The etiology of ED is multifactorial and can arise from vascular, neurological, hormonal, and psychogenic causes.^2^ Endothelial dysfunction and cardiovascular risk factors are among the primary causes, particularly in vasculogenic ED. While the diagnosis of vasculogenic ED can be made with penile Doppler ultrasonography (duplex ultrasound), the difficulties in routine and standardized application of this method make it difficult to use in practice.^3^ Its use in daily clinical practice is limited due to its invasive nature, the need for patient preparation, the risk of priapism, and radiologist dependency. Therefore, diagnosis is often made based on the patient’s clinical history, physical examination, and assessment of concomitant cardiovascular risk factors. Historically, the treatment of erectile dysfunction has passed many milestones.

Intracavernous agents, penile prosthesis implants, and the introduction of phosphodiesterase type 5 inhibitors (PDE5i) in 1998 are among the milestones in this process.^4^ However, long-term adherence to PDE5i and similar treatment regimens has been observed to be low.^5^ The main reasons for this include a reluctance to become dependent on medication, cost concerns, and the fact that these medications provide symptomatic, not curative, benefits. Considering these shortcomings, low-intensity shock wave therapy (Li-ESWT), introduced to the literature by Vardi et al. in 2010, is gaining interest as a non-invasive treatment alternative focused on physiological repair, particularly in vasculogenic ED.^6^

In this method, electrical energy is first converted into acoustic impulses and then into shock waves by a generator, thereby producing its biological effect. The biological effect begins with microbubbles created by the shock wave. These bubbles stimulate the endothelial wall and trigger NO release. This leads to smooth muscle relaxation and increased penile blood flow. In parallel, Li-ESWT improves cavernous perfusion by promoting angiogenesis and neovascularization via upregulation of growth factors, such as vascular endothelial growth factor.^7^ Currently, the European Urology Guidelines recommend Li-ESWT for mild to moderate vasculogenic ED.^8^ While the success rate of this method ranges from 60% to 80% across different series, the number of studies predicting treatment response is limited.^9^

In this study, we aimed to determine the factors that could predict Li-ESWT response in patients with ED by taking into account the demographic and clinical characteristics of the patients, using clinical history and various non-invasive data. Furthermore, based on the obtained predictor variables, we aimed to develop a simple, highly applicable, and objective scoring system that clinicians can easily use in daily practice and to introduce this system to the literature.

## Material and methods

### Study population

This prospective, non-randomized, interventional study was planned from January 2024 to January 2025. It included male patients aged 18-80 years, diagnosed with vasculogenic ED, classified as low- or moderate-risk according to the Princeton IV consensus criteria, who underwent Li-ESWT. In the present study, penile Doppler ultrasound was not routinely performed for either arteriogenic or venous etiology, reflecting current urological practice. Instead, the diagnosis of vasculogenic ED was placed with comprehensive evaluation of clinical and sexual history, physical examination, and laboratory findings. This evaluation excluded hormonal, neurogenic, and psychogenic etiologies that could cause ED. After these examinations, patients whose vascular risk factors and/or clinical history were suggestive of vascular genesis and/or with known clinical response to PDE-5 inhibitors of at least one were considered to have vasculogenic ED. Cardiovascular risk factors were defined as the presence of hypertension, dyslipidemia, current smoking, or obesity. Inclusion criteria were: treatment naïve for Li-ESWT, International Index of Erectile Function (IIEF)-5 EF score of 11-21, erection hardness score (EHS) of 2 or 3, regular sexual intercourse with partner, sex hormone levels within normal limits, and at least 6 months of stable ED. Patients with psychogenic ED, normal nocturnal penile tumescence, fully described morning erections, neurological disease, advanced heart failure, or severe cardiovascular disease, hypogonadism, radical prostatectomy, or pelvic radiotherapy, previous penile surgery, or Peyronie’s disease were excluded from the study. Patients with psychogenic ED were assessed using validated scales, including the Generalized Anxiety Disorder-7 (GAD-7) and the Patient Health Questionnaire-9 (PHQ-9), while taking sexual and clinical history. Patients were excluded from the study if their scores indicated clinically significant anxiety or depression, and psychiatric consultation was requested for these individuals. After applying the exclusion criteria, 219 patients were included in the study.

### Clinical information

Institutional ethics committee approval was obtained before the study (Ethics committee number: 17/757). The study was conducted using data from two centers. The following data were recorded: patient age, duration of ED, body mass index (BMI), response to previous PDE5i use, IIEF-5 EF domain score, EHS, comorbidities, and smoking history. All patients were referred for cardiology consultation. The radiologist then measured carotid intima-media thickness (cIMT) and brachial artery flow-mediated dilation rate (FMD), recording these measurements in the database. Patients using active PDE5i were included in the study after a 1-month washout period following discontinuation of treatment.

The results for endothelial activity were assessed with the help of brachial FMD that was non-invasive, reliable, and reproducible.^10^ The participants were given the opportunity to rest for at least 10 min before the measurements. The basal diameter of the brachial artery and the velocity of blood flow was then determined by duplex ultrasonography. A blood pressure cuff was inflated to 250 mmHg on the forearm, which led to ischemia in 5 min. It was followed by reactive hyperemia, and the highest arterial diameter was measured at 30 s and registered as the maximal diameter. The FMD ratio was determined by the formula; (maximum diameter − basal diameter)/basal diameter) × 100.

### Li-ESWT procedure

The same protocol was carried out in both centers to perform Li-ESWT. The therapy was done twice weekly, with 3000 pulses each time, for a total of 18 000 pulses administered over 3 weeks. Six sites were used bilaterally on the penis side lateral crura during each of the sessions (proximal, middle, and distal shaft). The procedure was performed using a Food and Drug Administration (FDA) -approved device with an electrohydraulic energy generator and a focusing probe (Modus ESWT, Inceler Medical, Ankara/Turkey). The device settings were 0.09 mJ/mm^2^ and 3 Hz, and individual sessions lasted about 15-20 min.

#### Clinical outcomes

Patients were called for follow-up at 1, 3, 6, and 12 months after treatment completion. At these visits, IIEF-5 EF scores and EHS were measured. Treatment success was assessed based on the results obtained at 6 months. A one-point increase in EHS or a five-point or greater increase in IIEF-5 EF scores was considered treatment response. Based on these criteria, patients were divided into responder and non-responder groups.

#### Creating the scoring system

**Figure 1:**
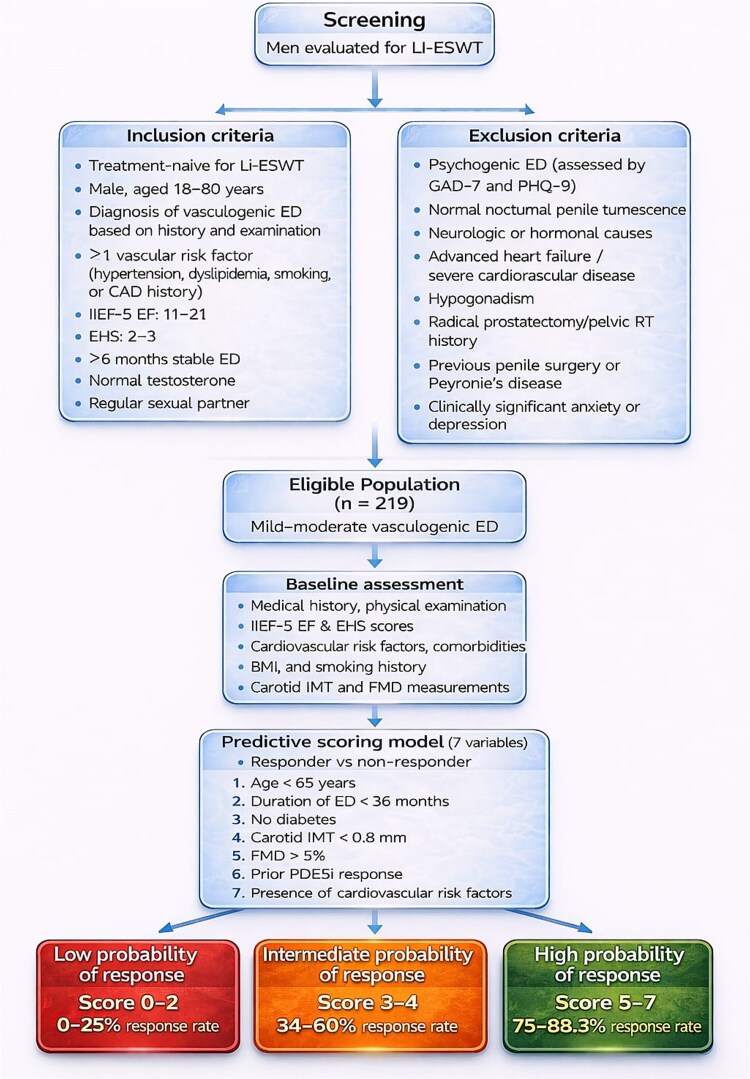
Clinical flowchart illustrating patient selection, baseline evaluation, development of the predictive scoring model, and stratification of treatment response probability.

Significant differences between responder and non-responder groups were examined using binary logistic regression analysis. Cut-off values for continuous variables were determined using receiver operating characteristic (ROC) curve analysis, and these values were used to convert variables into binary categorical variables. Each variable was scored based on beta coefficients and odds ratios in the regression model, and a new scoring system was developed accordingly. The flowchart and scoring system design are presented in Figure 1.

#### Sample size calculation

This prospective study included participants who all received the same treatment, with an expected increase of at least 5 points in the IIEF-EF score. Based on the published literature, a 70% response rate was estimated. To estimate this rate with 95% confidence and 10% margin of error, a minimum of 81 participants was required. Allowing for a 10% loss to follow-up, the final target sample size was set at 90.

### Statistical analysis

Continuous variables are reported as mean ± SD or median (IQR), and categorical variables are reported as numbers and percentages (%). Comparisons between groups were made using the student’s t-test for normally distributed continuous variables and the Mann–Whitney U test for non-normally distributed continuous variables. Categorical variables were analyzed using the chi-square or Fisher’s exact test. Variables predicted for inclusion in the scoring system were subjected to binary logistic regression analysis. The relationship between the obtained total scores and treatment success was tested using ROC curve analysis. The scoring system was calibrated using the Hosmer–Lemeshow test, and internal validation was performed using bootstrapping. All statistical analyses were performed using R software (version 4.4.2).

## Results

The mean age of the 219 patients included in the study was 53.4 ± 11.9 years, and the mean BMI was 27.6 ± 4.1 kg/m^2^. Fifty-five (25.1%) of the patients had diabetes mellitus (DM), 49 (22.4%) had coronary artery disease, and 153 (69.9%) had cardiovascular risk factors. The mean duration of ED was 35.8 ± 13.5 months. The mean pre-procedural IIEF-5 EF score was 16.3 ± 3.1, and the median EHS score was 2 (2-3). The treatment response rate 6 months after the Li-ESWT procedure was 66.2% (145/219). Patients were divided into 2 groups: responders (*n* = 145) and non-responders (*n* = 74) based on their treatment response. Pre-procedural clinical and demographic characteristics revealed a median age of 55 (IQR: 47-61) years in the responder group and 60 (IQR: 43-71) years in the non-responder group (*P* = .037). The presence of DM, history of PDE5i use, FMD rate, ED duration, and cIMT were statistically different between the groups (*P* < .01 for each variable). At the 6-month visit after Li-ESWT, the IIEF-5 EF score was 21.7 ± 3.5 points in the responder group and 18.2 ± 3.9 points in the non-responder group. The distribution of variables between the 2 groups is summarized in Table 1.

**Table 1:** Comparison of baseline and outcome variables between responders and non-responders.

Variable	Non-responders (*n* = 74)	Responders (*n* = 145)	*P* value
Age (years), median (IQR)	60 (43-71)	55 (47-61)	.037^a^
BMI (kg/m^2^), mean ± SD	27.6 ± 4.2	27.7 ± 4.1	.846^a^
CVD risk factors (+), *n* (%)	37 (50.0%)	116 (80.0%)	<.001^b^
CAD (+), *n* (%)	21 (28.4%)	28 (19.3%)	.118^b^
Diabetes mellitus (+), *n* (%)	31 (41.9%)	24 (16.6%)	<.001^b^
Previous PDE5i benefit (+), *n* (%)	36 (48.6%)	116 (80.0%)	<.001^b^
cIMT (mm), mean ± SD	0.81 ± 0.08	0.78 ± 0.04	.001^a^
FMD (%), mean ± SD	4.6 ± 2.8	6.1 ± 1.5	<.001^a^
ED duration (months), mean ± SD	41.9 ± 15.9	32.6 ± 11.1	<.001^a^
IIEF (pre-Li-ESWT), mean ± SD	16.8 ± 3.6	16.3 ± 2.9	.266^a^
IIEF (6th month), mean ± SD	18.2 ± 3.9	21.7 ± 3.5	<.001^a^
EHS (baseline), median (IQR)	2 (2-2)	2 (2-3)	.223^c^
EHS (6th month), median (IQR)	2 (2-2)	3 (2-3)	<.001^c^

a Independent samples t-test.

b Chi-square test.

c Mann-Whitney U test.

Abbreviations: BMI, body mass index; CVD, cardiovascular disease; cIMT, carotid intima-media thickness; ED, erectile dysfunction; EHS, Erection Hardness Score; FMD, flow-mediated dilation; IIEF, International Index of Erectile Function; Li-ESWT, low-intensity extracorporeal shockwave therapy; PDE5i, phosphodiesterase type 5.

Logistic regression analysis was performed with potential variables to predict treatment response. The regression model included variables that had statistically significant differences between the two groups. ROC analysis was used to assess FMD, cIMT, age, and ED duration, and to obtain cut-off values. These continuous variables were then turned into categorical variables depending on the values that were above the cut-off points, as well as those below the cut-off points. To fit the scoring system, cut-off values were rounded to the nearest whole number or to a clinically significant level. Accordingly, age was categorized as <65 years (derived from a cut-off of ≤63.5 years), erectile dysfunction duration as <36 months (derived from ≤34.5 months), cIMT <0.8 mm (derived from 0.765 mm), and flow-mediated dilation as ≥5% (derived from ≥4.8%). These categorized variables were subsequently used to construct the scoring system (Table 2). In addition to these variables, DM, cardiovascular disease (CVD) risk factors, presence of coronary artery disease, and PDE5i response were included in the established logistic regression model. In multivariate logistic regression analysis, independent predictors of Li-ESWT success were; no diabetes (odds ratio [OR] = 2.67; 95% confidence interval [CI] 1.24-5.72; *P* = .012), ED duration less than 36 months (OR = 2.23; 95% CI 1.10-4.53; *P* = .026), cIMT less than 0.8 mm (OR = 2.04; 95% CI 1.03-4.06; *P* = .042), previous PDE5i response (OR = 2.47; 95% CI 1.18-5.15; *P* = .016), FMD ≥5% (OR = 2.57; 95% CI 1.23-5.34; *P* = .012), being under 65 years of age (OR = 2.28; 95% CI 1.07-4.83; *P* = .032) and the presence of cardiovascular risk factors (OR = 2.23; 95% CI 1.06-4.70; *P* = .036). Coronary artery disease was not found to be statistically significant (*P* = .118) (Table 3).

**Table 2:** ROC-based dichotomization of continuous variables and determination of optimal cut-off values.

Variable	AUC (95% CI)	Cut-off	Sens	Spec	*P*
Age (years)	0.67 (0.60-0.74)	≤63.5	59.5	82.1	<.001
ED duration (months)	0.68 (0.61-0.76)	≤34.5	82.4	41.4	<.001
cIMT (mm)	0.70 (0.62-0.78)	<0.765	82.4	36.6	<.001
FMD (%)	0.74 (0.66-0.83)	≥4.8	89.0	60.8	<.001

Abbreviations: AUC, area under the curve; CI, confidence interval; cIMT, carotid intima–media thickness; ED, erectile dysfunction; FMD, flow-mediated dilation.

**Table 3:** Multivariate logistic regression analysis of predictors of response to low-intensity extracorporeal shock wave therapy in patients with erectile dysfunction.

Variables	Beta	OR (95% CI)	*P* value
Diabetes mellitus			
Yes (reference category)	–	–	–
No	0.980	2.67 (1.24-5.72)	.012
ED duration			
≥36 months (reference category)	–	–	–
<36 months	0.803	2.23 (1.10-4.53)	.026
cIMT			
≥ 0.8 mm (reference category)	–	–	–
<0.8 mm	0.713	2.04 (1.03-4.06)	.042
Previous PDE5i benefit			
No (reference category)	–	–	–
Yes	0.903	2.47 (1.18-5.15)	.016
FMD			
<%5 (reference category)	–	–	–
≥5%	0.943	2.57 (1.23-5.34)	.012
Age category			
≥65 years (reference category)	–	–	–
<65 years	0.822	2.28 (1.07-4.83)	.032
CVD risk factors			
No (reference category)	–	–	–
Yes	0.801	2.23 (1.06-4.70)	.036
Coronary artery disease			
No (reference category)	–	–	–
Yes	0.645	1.91 (0.85-4.28)	.118

Abbreviations: Beta, regression coefficient; cIMT, carotid intima-media thickness; CAD, coronary artery disease; CI, confidence interval; CVD, cardiovascular disease; DM, diabetes mellitus; ED, erectile dysfunction; FMD, flow-mediated dilation; PDE5i, phosphodiesterase type 5 inhibitor; OR, odds ratio.

**Figure 2:**
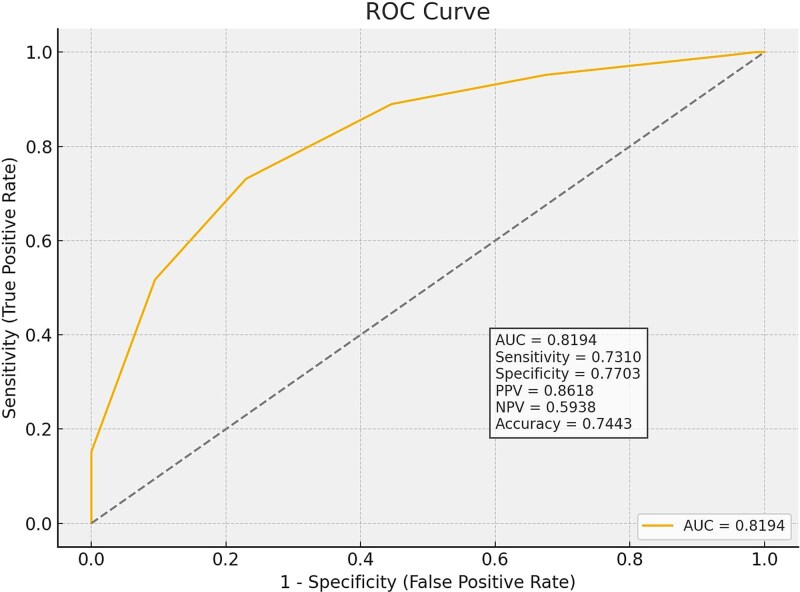
ROC curve for the total score predicting treatment response. Area under the curve is 0.819, indicating good discrimination. A cut-off of ≥4.5 provided sensitivity of 73.1% and specificity of 77.0%.

Based on the ORs and beta coefficients in this model, each variable was assigned a score of 0 or 1. The total score, calculated from the 7 variables, ranges from 0 to 7. High scores predict Li-ESWT success, while low scores predict treatment failure. The scoring system and its interpretations are shown in Table 4. ROC analysis revealed an area under the curve (AUC) of 0.819 (95% CI 0.762-0.877) for this new scoring system. With a cut-off value of 4.5 points, sensitivity was 73%, specificity was 77%, positive predictive value was 0.86, negative predictive value was 0.59, and overall accuracy was 74% (*P* < .001) (Figure 2).

**Table 4:** Predictive scoring system for Li-ESWT treatment response in erectile dysfunction.

Variable (1 point if present)	Score	Interpretation of total score:
CVD risk factors (Yes)	1	***0-2 points → Low response likelihood (0-25% response rate)*** ***3-4 points → Moderate response likelihood (34%-60% response rate)*** ***5-7 points → High response likelihood (75%-88.3% response rate)***
Age < 65 years	1	
FMD ≥ 5%	1	
Previous PDE5i benefit (Yes)	1	
cIMT <0.8 mm	1	
Diabetes mellitus (No)	1	
ED duration <36 months	1	

The total score ranges from **0 to 7 points**, calculated by summing 1 point for each variable present. Increasing total scores were associated with progressively higher response rates. For example, a 58-year-old patient with cardiovascular risk factors, preserved flow-mediated dilation (≥5%), previous benefit from PDE5i, absence of DM, and an erectile dysfunction duration <36 months would have a total score of **5 points**, corresponding to a **high likelihood of treatment response.**

Abbreviations: CVD, cardiovascular disease; cIMT, carotid intima-media thickness; ED, erectile dysfunction; FMD, flow-mediated dilation; PDE5i, phosphodiesterase type 5 inhibitor.

**Figure 3:**
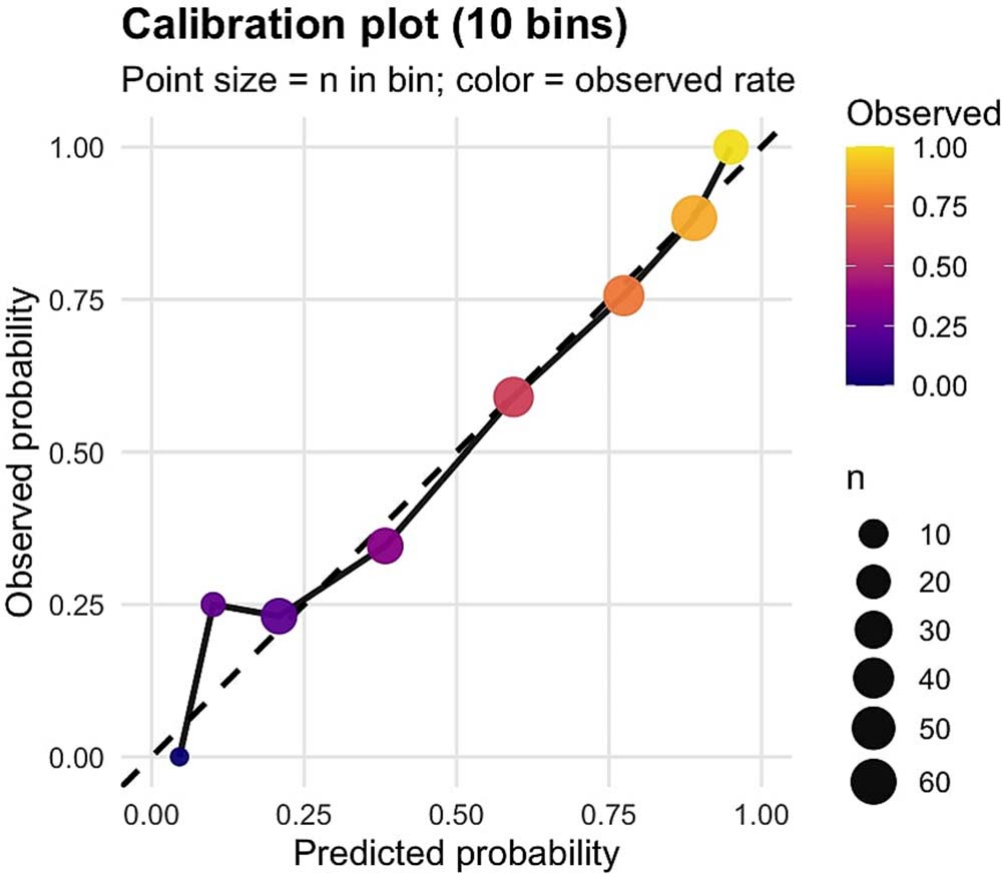
Calibration plot for the predictive scoring system using deciles of predicted risk.

The predicted and observed results showed good correlations in the calibration analysis. There was no indication that there was a lack of fit (Hosmer–Lemeshow goodness-of-fit test, (χ^2^ = 1.678, df = 4, *P* = .795) Bootstrap resampling (1000 iterations) internal validation showed that the models performed well, with good discrimination (AUC = 0.82; 95% CI 0.76-0.88) and no evidence of meaningful overfitting. The visual inspection of the calibration plot showed that the predicted and observed probabilities were very close across most risk strata, especially in the intermediate and high-risk strata (Figure 3).

## Discussion

In recent years, Li-ESWT has become an important treatment option for ED and is increasingly popular in clinical practice. Accurate prediction of treatment outcomes and personalized treatment planning are essential.^11^ Therefore, our study systematically examined clinical and vascular factors that may influence the success of Li-ESWT in vasculogenic ED and developed a corresponding scoring system. We found that the absence of DM, younger age (<65 years), shorter duration of ED (<36 months), positive response to PDE5i, FMD rate ≥ 5%, cIMT <0.8 mm, and the presence of CVD risk factors were independent predictors of positive outcomes. This scoring system, which assigns each of the 7 variables a value of 1, demonstrated sensitivity of 73% and specificity of 77% (AUC: 0.819) at a cut-off score of 4.5, indicating strong predictive power. This simple scoring system is expected to significantly enhance existing knowledge about predicting Li-ESWT results and aid in clinical decision-making.

Diabetes mellitus is a significant risk factor for ED, involving several pathophysiological mechanisms.^12^^,^^13^ Diabetic ED occurs due to impaired nitric oxide-mediated vasodilation, nerve damage, and hormonal changes caused by endothelial dysfunction.^12^^,^^13^ Diabetes mellitus contributes to both the development of ED and reduced treatment efficacy.^13^^,^^14^ In diabetic patients, Li-ESWT was shown to increase cavernous blood flow. Low-intensity extracorporeal shock wave therapy also enhances penile endothelial function. However, its effectiveness declines in severe or poorly-controlled DM. The therapeutic benefit is less long-lasting in diabetic men than in non-diabetic men.^15^ This study confirms that diabetes independently reduces treatment success. This is likely due to the complex causes of DM, including factors beyond vascular mechanisms and differences in diabetes duration and glycemic control.

Extensive literature documents that both patient age and duration of ED are key factors influencing Li-ESWT effectiveness.^16–18^ Beyond the impact of age-related health conditions, reduced vascular endothelial growth factor receptor activity has also been linked to weaker treatment responses.^19^ A prospective study of 52 patients showed that younger age (<45 years) and shorter ED duration (<2 years) are important predictors.^18^ Similarly, an analysis of 58 cases indicated that patients with an ED duration of ≤5 years experienced greater improvements in IIEF-EF scores compared to those with a duration of ≥10 years.^20^ Additionally, a prospective, non-randomized, single-arm study involving 56 patients found significantly poorer results in individuals aged ≥65 years and with 3 or more comorbidities, while those younger than 65 and with 2 or fewer comorbidities saw notable improvements at 1 and 6 months post-treatment.^16^ Consistent with current research, our study also identified that advanced age and longer ED duration are independent negative prognostic factors. Conversely, patients under 65 years old and with a disease duration of less than 36 months had significantly better treatment responses. The differences in cut-off values for age and ED length likely reflect variability in study populations.

Another key predictor of Li-ESWT success is the patient’s response to PDE5i therapy before starting Li-ESWT.^7^^,^^21^ Clinical data show that patients who improve erectile function after PDE5i treatment tend to have higher success rates with subsequent Li-ESWT, while those with moderate to severe ED who do not respond to PDE5i often experience less benefit.^21^ In a pooled analysis of 5 double-blind, placebo-controlled trials with 350 PDE5i responders and 48 non-responders, the lower effectiveness of Li-ESWT in non-responders was linked to more advanced cavernosal damage.^21^ Specifically, the PDE5i-responder group showed a notable rise in IIEF-EF scores 3 weeks after the sixth Li-ESWT session, with treatment success rates of 79.5% among responders and 55.4% among non-responders (*P* = .005).^21^ Supporting these results, our study found that a prior positive response to PDE5i is a strong predictor of Li-ESWT success in patients with vasculogenic ED. The common endothelial mechanisms involved in both therapies may explain this connection.

Flow-mediated dilation is a valuable parameter indicating endothelial function and nitric oxide-mediated vasodilation capacity. This parameter was applied to the penile artery in Li-ESWT studies and used to objectively observe changes in penile hemodynamics.^6^^,^^22–24^ In a series of 20 patients with ED for an average of 35 months, penile dorsal artery blood flow measured after 12 treatment sessions increased from 7.3 to 17.8 mL/min/dL at rest and from 12.0 to 28.9 mL/min/dL after occlusion, and spontaneous erections were reported in half of the patients (*P* < .001).^6^ Applying this method to the penile artery presents technical challenges. In contrast to such studies, our study established endothelial functioning by measuring FMD rate across the brachial artery prior to treatment, which indicates that it is an important predictor of Li-ESWT response.

Carotid intima-media thickness is considered an important risk factor both in atherosclerosis and cardiovascular events, and meta-analytic studies also established that cIMT is closely linked with ED severity.^25^^,^^26^ Increased cIMT values have been observed in men with no clinical evidence of atherosclerosis but with clinical vascular risk factors including diabetes, hypertension, or hyperlipidemia. Notably, individuals with cIMT ≥1.0 mm possess an approximately 2.6-fold greater risk of developing severe ED.^27^ The literature further suggests that cIMT may be correlated not only with disease severity but also with the response to PDE5i therapy among patients with vasculogenic ED.^28^^,^^29^ In a prospective cohort of 51 patients with vasculogenic ED, mean cIMT was significantly higher among tadalafil non-responders (0.9 ± 0.2 mm vs. 0.6 ± 0.2 mm, *P* = .000), with 90% of non-responders exhibiting cIMT values above 0.67 mm.^29^ Our study extends these findings by demonstrating that cIMT functions as an independent predictor of Li-ESWT efficacy, thereby implying that this parameter is associated not only with ED severity and PDE5i response but also with therapeutic outcomes following Li-ESWT.

Previous studies reported that coronary artery disease risk factors such as age, diabetes, hypertension, smoking, obesity, and hyperlipidemia are among the prognostic variables that negatively affect Li-ESWT success.^11^^,^^17^^,^^30^ However, there are also studies in which hypertension, diabetes, or hyperlipidemia alone are not associated with ED, and uncontrolled diabetes and uncontrolled hyperlipidemia are significantly associated with ED.^16^ Among these factors, only uncontrolled hyperlipidemia is an independent predictor of a negative Li-ESWT response.^17^ In this study, coronary artery disease was not found to be an independent predictor of treatment response. However, cardiovascular risk factors were found to be significant. This result suggests that endothelial dysfunction at the microvascular level, rather than macrovascular pathologies, may play a more critical role in the efficacy of Li-ESWT. Coronary artery disease is an advanced vascular pathology with a heterogeneous course, reflecting clinically evident coronary atherosclerosis. However, the mechanism of action of Li-ESWT is primarily based on angiogenesis, neovascularization, and improvement of endothelial function. This result suggests that the efficacy of Li-ESWT decreases in advanced vascular disorders.

Our study has several limitations. First, although the same protocol was applied, the Li-ESWT procedure was performed by 2 different operators. The presence of 2 different operators at 2 centers could lead to selection bias. However, this was minimized by applying the same treatment protocol. Second, our study did not have any sham or control groups that were not randomized. Another important limitation is the lack of objective hemodynamic confirmation via penile Doppler ultrasound in all patients. It should be noted that our study population was defined by rigorous clinical diagnosis rather than invasive vascular assessment. Consequently, the developed scoring system predicts clinical response to Li-ESWT across a broad spectrum of vasculogenic ED—including microvascular and endothelial dysfunction—rather than focusing solely on pure arteriogenic ED. We believe this clinical approach better reflects real-world urological practice, where non-invasive diagnostic tools are prioritized for routine evaluation. Finally, and perhaps most importantly, the major limitation is the lack of external validation of the scoring system. Future studies should validate this model in independent, multicenter cohorts with different patient populations, ideally including different Li-ESWT devices and treatment protocols.

## Conclusion

Our study provides a significant contribution to the literature by developing the first scoring system to predict Li-ESWT response in patients with vasculogenic ED. It is believed that his system, based on non-invasive and easily applicable parameters, will be a powerful aid in treatment decision-making in clinical practice. However, the model requires validation in prospective, randomized studies with larger samples and diverse patient populations.
